# Glutathione Transferase (GST)-Activated Prodrugs

**DOI:** 10.3390/pharmaceutics5020220

**Published:** 2013-04-02

**Authors:** Paolo Ruzza, Andrea Calderan

**Affiliations:** Institute of Biomolecular Chemistry of CNR, Padova Unit, Via Marzolo 1, Padova 35131, Italy; E-Mail: andrea.calderan@unipd.it

**Keywords:** glutatione transferase, glutathione, anticancer prodrugs

## Abstract

Glutathione transferase (formerly GST) catalyzes the inactivation of various electrophile-producing anticancer agents via conjugation to the tripeptide glutathione. Moreover, several data link the overexpression of some GSTs, in particular GSTP1-1, to both natural and acquired resistance to various structurally unrelated anticancer drugs. Tumor overexpression of these proteins has provided a rationale for the search of GST inhibitors and GST activated cytotoxic prodrugs. In the present review we discuss the current structural and pharmacological knowledge of GST-activated cytotoxic compounds.

## 1. Introduction

For over 50 years chemotherapy has been used with varying success in the treatment of metastatic cancers. The major problems with the use of many chemotherapeutic agents are their unacceptable damage to normal cells and organs, a narrow therapeutic index, a relatively poor selectivity for neoplastic cells, and multidrug resistance upon prolonged treatment due to up-regulation of efflux pumps, increased glutathione transferase (GST; EC 2.5.1.18) expression, and enhanced DNA repair [1,2,3,4]. A potential strategy to overcome these limitations is the use of prodrugs. A more rational approach to design prodrugs is based on molecular targets that are responsible for cell transformation. Prodrugs may be divided into two groups: (1) molecules designed to improve the bioavailability and the pharmacokinetic of antitumor agents and (2) compounds designed to locally deliver antitumor agents.

## 2. Glutathione and Glutathione Transferase

Glutathione (γ-l-glutamyl-l-cysteinylglycine, GSH) is widely distributed in animal tissues, plants, and microorganisms. It is typically present in high cellular levels (0.1–10 mM). In many cells, GSH accounts for more than 90% of the total nonprotein sulfur. The two characteristic structural features of GSH (γ-Glu linkage, and the SH group) promote its intracellular stability and are intimately associated with its functions: it participates in reactions involving the synthesis of proteins and nucleic acids and in those that detoxify free radicals and peroxides.

**Figure 1:**
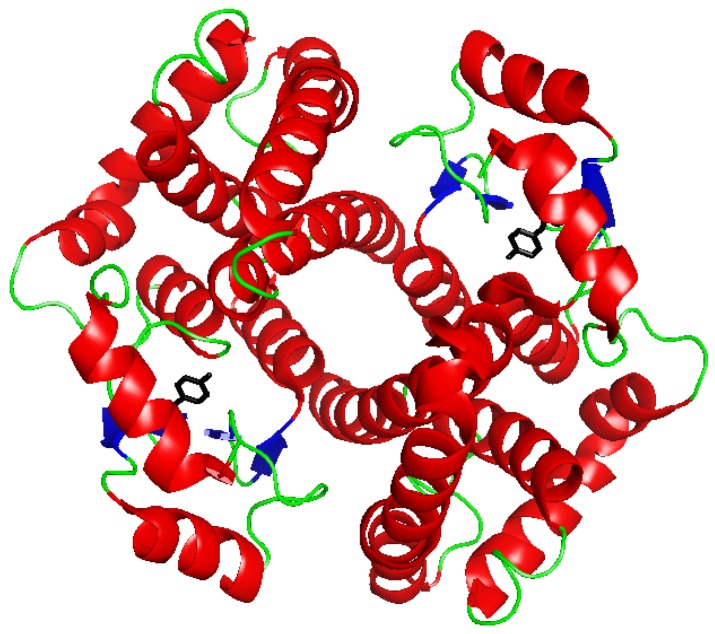
Ribbon diagram of human GSTP1-1 (PDB file 1PKW) [15]. In black the Tyr residue initially proposed as involved in the activation of glutathione (GSH).

GSTs constitute a superfamily of dimeric proteins (Figure 1) that catalyze the conjugation of the tripeptide GSH to electrophiles resulting in the formation of the corresponding GSH conjugates [5,6,7]. These proteins are found in almost all species and are divided into classes, based primarily on sequence similarity. Currently recognized classes of cytosolic GSTs in mammals include the Alpha, Mu, Omega, Pi, Sigma, Theta and Zeta classes [8]. GSTs from the last two classes have also been identified in plants and other organisms. Other distinct groups have been identified only in insects (Delta and Epsilon [9]), plants (Lambda, Phi and Tau [10]) or prokaryotes (Beta [11]). A Kappa class regroups mitochondrial GSTs, and an independent group of proteins, called “membrane-associated proteins involved in eicosanoid and glutathione metabolism” (MAPEG), are integral membrane components in microsomal and mitochondrial cell fractions with GSH conjugating activities similar, but structurally unrelated, to the soluble GSTs [8]. In mammals, GSTs are present in virtually all tissues. In rats, high levels of both cytosolic and membrane-bound GST activities have been found in the liver [12]. In humans, the highest cytosolic GST activity level is present in the liver, whereas the kidney (78% of liver), lung (34% of liver), and intestine (37% of liver) have lower activity levels [13].

The three-dimensional structures of several classes of soluble GSTs have been resolved by X-ray crystallography. The quaternary structure of human GSTP1-1 is showed in Figure 1. Each GST subunit is composed of an *N*-terminal and a *C*-terminal domain. This last domain, that adopts an α-helix structure, contains a portion of the GSH-binding site (G-site) and a great part of the binding site for hydrophobic electrophiles (H-site). The *N*-terminal domain adopts a thioredoxin-like fold (βαβαββα) and comprises most of the G-site. This domain is quite conserved among the different GST classes, whereas the *C*-terminal domain is more divergent. Variations in hydrophobic amino acid residues of the H-site are strongly related to substrate selectivity [14].

The G-site of most soluble GSTs contains a Tyr or Ser residue (e.g., Tyr7 in the Pi class, and Ser11 in the Theta class) located within hydrogen bonding distance from the sulfhydryl group of the enzyme-bound GSH. When the tyrosine is substituted by a phenylalanine, the catalytic activity is dramatically reduced [16,17], so it has been suggested that tyrosine, acting like a base, could receive the proton from the GSH thiol group, thereby activating it [18,19]. Successive studies point to the fact that the GSH glutamyl α-carboxylate group is essential for GSH activation [20], whereas others indicate the importance of the active-center water molecules [21]. Recently, Dourado *et al.* [22] proposed a water-assisted proton-transfer mechanism that integrates the suggested roles of the GSH glutamyl α-carboxylate group and the active-center water molecules in GSH activation. After an initial conformational rearrangement of GSH, a water molecule, acting as a bridge, is able to transfer the proton from the GSH thiol group to the GSH glutamyl α-carboxylate group.

Some GSTs (e.g., GSTO1-1) have, instead of Tyr or Ser, a Cys in their G-site that makes a mixed disulfide with GSH [23]. These enzymes have generally poor conjugative activity towards typical GST substrates, and seem instead implicated in redox reactions [23,24].

Class Pi GST (P1-1) is frequently overexpressed in rat and human tumors, including carcinoma of the colon, lung, kidney, ovary, pancreas, esophagus, and stomach [25,26,27]. Overexpression of class Pi GST in breast cancer and renal cell carcinoma can be used as a significant prognostic factor of these diseases. Although GSTs’ detoxifying activity protects cells from certain diseases, unfortunately it also reduces the effectiveness of certain chemotherapeutic drugs against cancer cells. Indeed, certain alkylating agents used in antineoplastic therapy belong to the classes of electrophilic compounds that are substrates for the GSTs [27,28]. A possible origin for the appearance of chemotherapeutic resistant tumor cells that no longer respond appropriately to antineoplastic agents, referred to as multi-drug resistance, may be an increase in the expression of total GST activity in tumor cells [29,30]. A plausible mechanism by which GSTs could contribute to drug resistance includes GST-dependent prevention of drug-induced apoptosis via direct interaction with signal transduction proteins, as suggested for GSTP1-1 [31,32] which inhibits c-Jun *N*-terminal kinase.

Consequently, the development of GST inhibitors appears as a rational response to overcome multi-drug resistance, increasing the effectiveness of antitumor drugs. A recent review has been published summarizing the progress in the development of GST inhibitors [33]. On the other hand, the overexpression of some GST enzymes by several tumors makes them a promising target for the prodrug therapy of cancer. GST-activated prodrugs are latent cytotoxic GST ligands, which undergo GST-catalyzed and GSH-dependent or -independent breakdown to release a cytotoxic species responsible for their anticancer effect. In this review we discuss the current structural and pharmacological knowledge of GST activated cytotoxic compounds.

### 2.1. GSH-Dependent Prodrugs Activation

**Figure 2:**
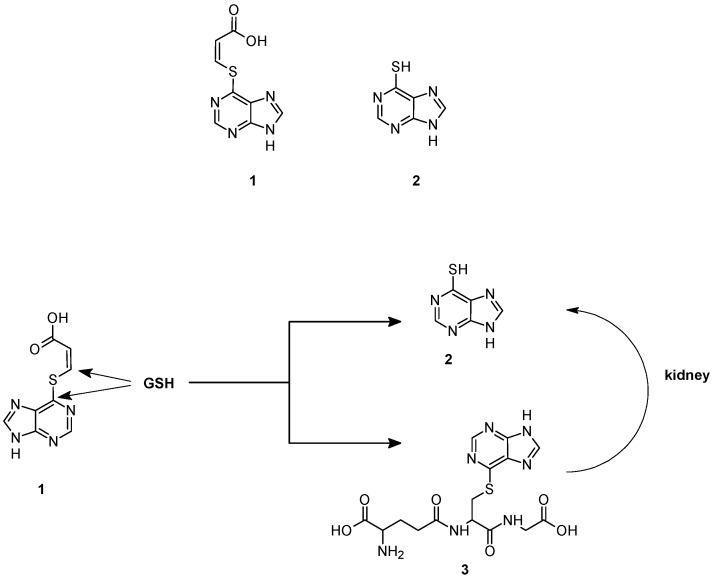
Chemical structure and mechanism of activation of *cis*-3-(9*H*-purin-6-ylthio) acrylic acid (**1**).

A first example of GSH-dependent prodrug activation has been reported by Gunnarsdottir and Elfarra [34]. They found that *cis*-3-(9*H*-purin-6-ylthio) acrylic acid (**1**) a prodrug of the cytotoxic agent 6-mercaptopurine (**2**), releases the active drug upon reaction with GSH, both *in vitro* and *in vivo*, via two different pathways, as shown in Figure 2. 6-Mercaptopurine can be originated indirectly through the formation and the further metabolism of the GSH adduct **3**, obtained by the nucleophilic attack of GSH on the C-6 carbon of the purine ring. The addition of rat liver homogenate, purified rat liver GSTs, or human recombinant GSTs did not increase the rate of this reaction suggesting that it occurs through a non-enzymatic mechanism. The adduct **3** is converted to 6-mercaptopurine by the sequential action of renal γGT dipeptidase and cysteine *S*-conjugate β lyase. In the second pathway, 6-mercaptopurine is formed directly from *cis*-3-(9*H*-purin-6-ylthio) acrylic acid which acts as a Michael acceptor and undergoes an addition-elimination reaction by attack of GSH to the β-carbon of the acrylic acid moiety. Rat GSTs and human recombinant GSTA1-1, GSTM1-1 and GSTP1-1 catalyze the reaction [35], albeit the direct formation of 6-mercaptopurine from prodrug **1** was found to occur to some extent non-enzymatically. Collectively, these data suggest that *cis*-3-(9*H*-purin-6-ylthio) acrylic acid targets tumors with up-regulated levels of GSH, GST or γGT, an enzyme overexpressed in several human cancers [36].

**Figure 3:**
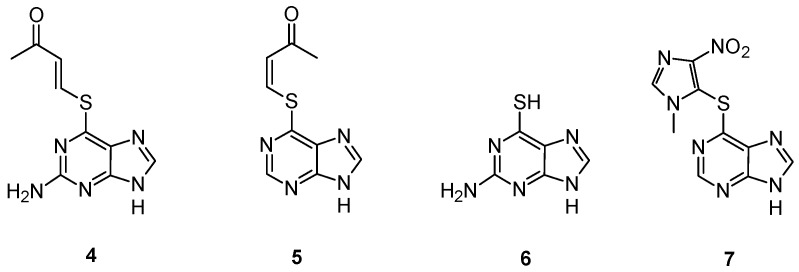
Chemical structure of compounds **4**, **5**, **6** and **7**.

The slow rate of activation of this prodrug, observed both *in vitro* and *in vivo*, induced Gunnarsdottir and Elfarra to explore the properties of α,β-unsaturated structural analogs of **1**, having the sulfur heteroatom conjugated to a butenone moiety. These analogs, lacking in an ionizable group at physiological pH, were expected to react more efficiently with GSH than **1** to yield the cytotoxic thiopurine [34]. Two compounds, namely *trans*-6-(2-acetylvinylthio)-guanine (**4**) and *cis*-6-(2-acetylvinylthio)-purine (**5**) (Figure 3), respectively, have been extensively studied [37]. In cell culture, the uptake and conversion to active 6-thioguanine (**6**) was associated with a concomitant decrease in cellular GSH levels, while a lower intracellular 6-thioguanine level was observed in GSH-depleted cells than in cells having intact GSH levels. An extensive assessment of the cytotoxicity of the two prodrugs revealed that both compounds **4** and **5** are generally more cytotoxic than the corresponding parent thiopurine, exhibiting a remarkable growth-inhibitory activity towards leukemic and melanoma cells [38].

Myelotoxicity studies in mice showed no reduction of circulating white blood cells upon administration of both compounds, whereas animals treated with equimolar doses of **6** showed a significant leukopenia [37]. The rates of GSH-mediated activation of compounds **4** and **5** are enhanced by some human GSTs including GSTM1-1 [39], frequently overexpressed in tumor cells with acquired resistance to nitrogen mustards [40], while GSTP1-1 had lower or not detectable activity towards these compound. Thus, these thiopurine prodrugs might be conceivably useful for treatment of tumors with upregulated levels of GSH and/or specific GSTs.

GSTs catalyzed also the activation of azathioprine (**7**) (Figure 3) [41], a 1-methyl-4-nitroimidazol-5-yl derivative of 6-mercaptopurine used for immunosuppression in connection with organ transplantation and in the treatment of autoimmune diseases [42]. This molecule showed a significant nonenzymatic reaction with GSH, even if the major fraction of azathioprine is bioactivated by the GSTs in the liver [41]. These data may explain the adverse drug reactions observed in the clinical use of azathioprine that could be related not only to the thiopurine *S*-methyltransferase (TPMT) phenotype, as generally assumed, but also to excessive liberation of 6-mercaptopurine and GSH depletion as results of high GST activities [41].

**Figure 4:**
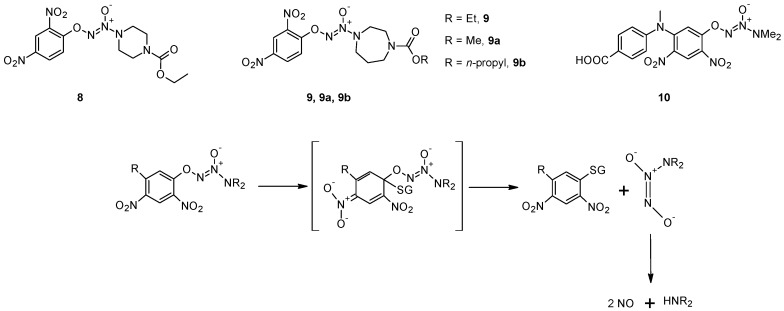
Chemical structures of compounds **8**, **9** and **10**, and mechanism of nitric oxide release.

Shami *et al.* [43] designed a library of arylated diazeniumdiolates as prodrugs for NO release by reaction with cellular thiols such as GSH, with or without catalysis by GST. Among the various compounds tested, O^2^-(2,4-dintrophenyl)-1-[(4-ethoxycarbonyl)piperazin-1-yl]diazen-1-ium-1,2-diolate (**8**) is the most active both *in vitro* and *in vivo* [43]. In these compounds, the authors exploited the well-known GST-catalyzed reaction between 1-chloro-2,4-dinitrobenzene and GSH, in which a Meisenheimer complex is formed before the elimination of the Cl^−^ anion. In the case of compound **8** the Cl^−^ anion is replaced by a diazeniumdiolate that spontaneously decomposes, producing two equivalents of NO (Figure 4). Molecular modeling performed using the Meisenheimer complex of **8** as GST ligand indicated that it could be accommodated in the catalytic sites of GSTM1-1 and A1-1, while steric constraints counteract its accommodation in the catalytic site of GSTP1-1, as confirmed by the evaluation of the NO release from this prodrug. *In vitro* studies showed that this molecule inhibits, in concentration-dependently mode, the growth of HL-60 and U937 human leukemia cells with activity in the submicromolar range. This prodrug has been found to be active also against human prostate (PPC-1), and multiple myeloma (OPM1) xenografts in mice [43,44,45]. Chakrapani and co-workers synthesized a number of structural analogues of **8** and their chemical and biological properties were compared with those of the parent compound [46]. Compound **9** (Figure 4) displays comparable anti-cancer activity to that of **8** in a number of cancer cell lines, but with a diminished reactivity towards GSH and GSH/GST that may be advantageous in the development of this class of anti-cancer agents. In addition, this molecule, as well as compound **8**, is selectively toxic towards renal cancer cell lines at concentrations that does not significantly affect the proliferation of normal renal epithelial cells [46].

The molecular modeling of the Meisenheimer complex indicated that replacing the piperazine ring with a smaller amino group might improve its accommodation in the catalytic site of GSTP1-1. On the other hand, the presence of a sterically bulk substituent at the 5-position of the 2,4-dinitrophenyl ring should diminish its suitability as a GSTA1-1 ligand, but, at the same time, improve its accommodation in the active center of GSTP1-1. Based on these considerations, Findlay and co-workers developed O^2^-[2,4-dinitro-5-(*N*-methyl-*N*-4-carboxyphenylamino) phenyl] 1-*N*,*N*-dimethylamino)diazen-1-ium-1, 2-diolate (**10**), an NO-releasing prodrug which was found to be efficiently metabolized by GSTP1-1 but not GSTA1-1, validating the molecular modeling predictions [47]. This molecule significantly delayed tumor growth when administered to immunodepressed mice bearing A2780 human ovarian carcinoma xenografts, its activity being comparable to that of cisplatin, which is the standard of care for management of ovarian cancer [47].

**Figure 5:**
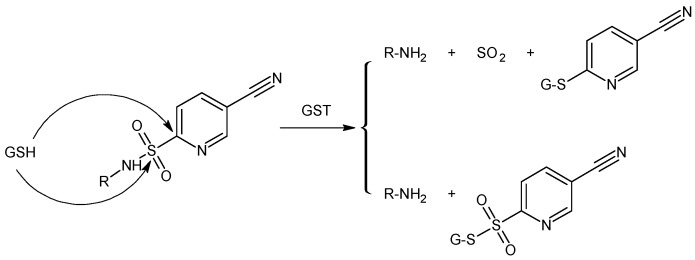
Proposed mechanistic pathway for glutathione transferase (GST)/GSH-mediated sulfonamide cleavage.

GST exhibits also a sulphonamidase activity that catalyzes the GSH-mediated hydrolysis of sulphonamide bonds to form the corresponding amine [48,49]. Zhao *et al.* [48] have shown that GST-mediated sulphonamide cleavage is relatively independent of the nature of the amine derivative, suggesting that any amine, in principle, can be derivatized by linkage with sulphonyl moieties, modifying the degree of intracellular liability (Figure 5). Recently, Axarli and co-workers [50] used this approach to synthesise a peptidyl-derivative activated intracellularly by the action of GSTA1-1. This chimaeric prodrug featured a bombesin peptide analogue that is specifically recognized by tumor cell specific receptor, providing a vehicle for selective drug delivery. The GST-mediated cleavages of the sulfonamide bond induced the release of GST inhibitor.

### 2.2. GSH-Independent Prodrugs Activation

**Figure 6:**
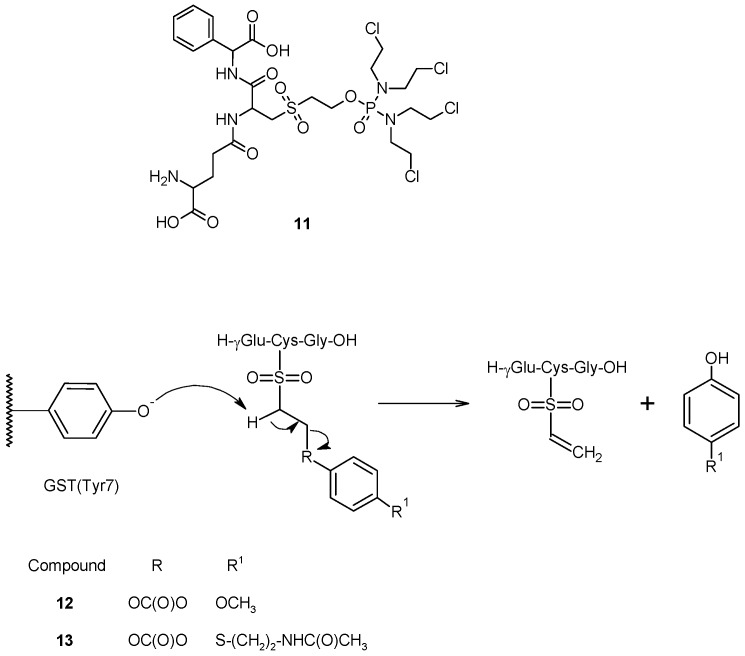
Chemical structure and mechanism of activation of compounds **11**, **12** and **13**.

A similar approach, involving the cleavage of sulfone bond, was pursued in the design of TLK286 (**11**) a GST-activated prodrug which consists of a GSH analog backbone (γ-Glu-Cys-Phg), linked to a tetrakis(chloroethyl)phosphorodiamidate moiety (Figure 6) through a sulfone linkage [51,52]. This prodrug is metabolized by GSTP1-1 to an alkylating nitrogen mustard and a vinyl sulfone, both of which are released intracellularly. In this molecule, the cytotoxic agent was conjugate to a GSH analog by the thiol group, which was successively oxidized to sulfone. The Tyr7 in the G site of GSTP1-1, responsible for GSH activation, might abstract a proton from the carbon adjacent (α-carbon) to the sulfone group of the resulting compound, causing a β-elimination reaction and the release of a cytotoxic species [52]. Early *in vitro* experiments revealed that the cleavage of TLK286 is efficiently catalyzed by both human GSTP1-1 and GSTPA1-1 [53], and results in two fragments, a phosphoroamidate nitrogen mustard that can alkylate cellular nucleophiles, including DNA, and a vinyl sulfone. Further preclinical studies have shown that tumor sensitivity to TLK286 positively correlates with GSTP1-1 expression both in cell cultures and in human tumor xenograft models, and that treatment with therapeutic doses of the drug produces only mild bone marrow toxicity in mice [54,55,56].

Recently, based on this structure, we proposed a prodrug activated sequentially by GST and then by tyrosinase towards metastatic melanoma [57]. In this molecule, the tyrosinase activated melanoma prodrugs (4-methoxyphenol and *N*-acetyl-4-*S*-cysteaminylphenol) were linked to GSH by an ethoxycarbonyl moiety, and the Cys sulfhydryl group was oxidized to sulfone to give compounds **12** and **13** (Figure 6). In this way, the Tyr phenoxide present in the G-site of GST would be able to abstract one of the acidic methylene protons linked to the sulfone moiety, releasing the tyrosinase-activated melanoma prodrugs. Preliminary *in vitro* studies revealed that these compounds were cleaved much more quickly in incubates containing liver or kidney cytosolic fractions, rich in GSTs, than in phosphate buffer, confirming the catalytic effect of this enzyme.

## 3. Conclusions

Resistance of various human tumors to cancer chemotherapeutic agents has been directly correlated with overexpression of some GST enzymes, and their capability to catalyze the drug conjugation to GSH. These observations have provided the development of low-molecular weight GST-inhibitors as tumor chemosensitizers [33] and, more important, the design and synthesis of latent cytotoxic prodrugs, GST-catalyzed and GSH-dependent or -independent activated, with promising results in preclinical studies. A further development may result from the recent discovery that some GSTs interact with kinases involved in controlling stress response, apoptosis and proliferation. In particular, GSTP1-1 has been characterized as an inhibitor of JNK1, a kinase implicated in the apoptotic response to cytotoxic stimuli. Identification of the location and nature of JNK1-GSTP1-1 interaction may conceivably lead to effective tumor chemosensitizers.
